# Unveiling the Role of DNA Methylation in Kidney Transplantation: Novel Perspectives toward Biomarker Identification

**DOI:** 10.1155/2019/1602539

**Published:** 2019-01-15

**Authors:** Antonella Agodi, Martina Barchitta, Andrea Maugeri, Guido Basile, Matilde Zamboni, Giulia Bernardini, Daniela Corona, Massimiliano Veroux

**Affiliations:** ^1^Department of Medical and Surgical Sciences and Advanced Technologies “GF Ingrassia”, University of Catania, Via S. Sofia, 87, 95123 Catania, Italy; ^2^Department of General Surgery and Medical-Surgical Specialties, University of Catania, Via Plebiscito, 628, 95124 Catania, Italy; ^3^Vascular Surgery Unit, University Hospital of Catania, Via Santa Sofia 87, 95123 Catania, Italy

## Abstract

The burden of chronic kidney disease is dramatically rising, making it a major public health concern worldwide. Kidney transplantation is now the best treatment for patients with end-stage renal disease. Although kidney transplantation may improve survival and quality of life, its long-term results are hampered by immune- and/or non-immune-mediated complications. Thus, the identification of transplanted patients with a higher risk of posttransplant complications has become a big challenge for public health. However, current biomarkers of posttransplant complications have a poor predictive value, rising the need to explore novel approaches for the management of transplant patient. In this review we summarize the emerging literature about DNA methylation in kidney transplant complications, in order to highlight its perspectives toward biomarker identification. In the forthcoming future the monitoring of DNA methylation in kidney transplant patients could become a plausible strategy toward the prevention and/or treatment of kidney transplant complications.

## 1. Introduction

The burden of chronic kidney disease (CKD), in terms of human suffering and economic costs, is dramatically rising, making it a major public health concern worldwide [1]. The management of end-stage renal disease (ESRD) patients requires life-saving dialysis or kidney transplantation. Patients receiving renal replacement therapy are appraised at more than 1.4 million worldwide, with an estimated ≈8% increasing incidence each year [2]. The main reasons of this raise are ageing of populations and the consequent increasing incidence of type 2 diabetes mellitus and hypertension, which are the key risk factors for CKD [3].

Kidney transplantation currently remains the best replacement therapy for patients with irreversible ESRD [4], since it is associated with improved survival and quality of life compared to hemodialysis [5], but either immune- or non-immune-mediated complications significantly contribute to the higher morbidity of transplant patients [5]. While short-term kidney graft survival after transplantation has continuously improved over recent years [5], current evidence reports less marked improvements in long-term outcomes [6–10]. Several factors may affect transplant outcomes, including donor age, alloimmune response, ischemia-reperfusion injury, interstitial fibrosis of the allograft, recipient comorbidity, degree of human leukocyte antigen mismatch and polymorphisms in immunologic and nonimmunologic genes [11–14]. More recently, a particular consideration has been given to genomic and epigenomic differences between the donor and the recipient, which encompass 3.5 to 10 million genetic variants and substantial epigenetic variations related to ethnicity, environment, and lifestyles [15–17]. Blood-based biomarkers have been widely proposed as potential predictive and diagnostic biomarkers, allowing the early identification of patients at high risk of transplant rejection and other adverse outcomes. The study of epigenetic mechanisms—including DNA methylation, histone modification, and noncoding RNA—is getting a lot of interest in this field of research, as reported by previous reviews [18–20].

In this review we provide an overview on how DNA methylation affects development and progression of CKD and we summarize the emerging literature about DNA methylation in kidney transplant complications. Finally, we discuss the perspectives and the clinical usefulness of DNA methylation changes as biomarkers of kidney transplant complications.

## 2. Epigenetics

Epigenetic mechanisms regulate gene expression without altering the DNA sequence. These molecular processes characterize the epigenome, which is dynamic in response to environmental stressors, modifiable during cell differentiation, and heritable in daughter cells [21]. There are several epigenetic mechanisms, which have been extensively reviewed by Portela and Esteller [22], affecting chromatin condensation, thereby regulating gene expression [23]: histone modifications (e.g., methylation or acetylation), noncoding RNA (e.g., siRNAs, lncRNAs, miRNAs), and DNA methylation [24].

The first lines of evidence on the role of epigenetics have been pointed out by cancer research, with several studies and meta-analyses demonstrating that epigenetic mechanisms regulate tumour suppressor genes silencing, activation of oncogenes, and increased chromosomal instability [25–29]. DNA methylation almost exclusively occurs within CpG islands—short sequences in gene promoters and regulatory regions that typically contain about 5-10 CpG dinucleotides per 100 bp [30]. In mammals, DNA methylation process is mediated by the activity of three DNA methyltransferases (DNMT1, DNMT3a, and DNMT3b).

## 3. DNA Methylation and Chronic Kidney Disease

Aberrant DNA methylation has been also described in other chronic diseases, such as cardiovascular disease, neurodegenerative diseases, diabetes and its complications, obesity, and CKD [31–38]. The latter has been recently associated with changes in the DNA methylation profile by* in vivo* and epidemiological studies [39]. Evidence from animal models indicated that* in utero* restriction of calories, proteins, and oxygen was linked to reduced nephron number, hypertension, and microalbuminuria. The influence of the intrauterine environment on the foetal epigenetic programming might explain foetal origin of adult diseases [40–42]. Beyond developmental programming, metabolic changes might also affect CKD development and long-term health. For instance, epidemiologic studies showed that the hyperglycaemia-related risk of diabetic kidney disease persisted even when metabolic control was restored. The discovery of the long-lasting effect of hyperglycaemia was the breakthrough for the development of the “metabolic memory” theory, particularly in the context of diabetic nephropathy [43, 44]. Consistently, the comparison between saliva samples of diabetic patients with or without end-stage kidney disease identified 187 genes that were differentially methylated, out of which 39 were involved in kidney development or diabetic nephropathy [45].

Recently, Smyth and colleagues compared DNA methylation of 485,577 CpG sites in blood samples between 255 CKD patients and 152 healthy controls [46]. Interestingly, they found aberrant DNA methylation of genes with known biological function in CKD (i.e., CUX1, ELMO1, FKBP5, INHBA-AS1, PTPRN2, and PRKAG2 genes). The relationship between PRKAG2 and CKD has also been confirmed by a meta-analysis of genomewide association data [47]. In addition, the Chronic Renal Insufficiency genomewide study compared the DNA methylation profiles of blood samples among patients classified by different glomerular filtration rates. The authors identified several differentially methylated regions in genes that were associated with kidney functions, including those involved in the epithelial to mesenchymal transition pathway (i.e., NPHP4, IQSEC1, and TCF3) [48].

Several lines of evidence also suggested the role of DNA methylation in kidney fibrosis progression. Stenvinkel and colleagues analysed DNA methylation of blood samples in CKD patients to evaluate the association between renal function, surrogate markers of inflammation, and aberrant DNA methylation. The authors concluded that stable CKD patients with no evidence of inflammation had comparable DNA methylation levels with age- and sex-matched controls, while end-stage kidney disease patients with higher inflammation exhibited DNA hypermethylation [49].

## 4. DNA Methylation in Kidney Transplantation

The identification of subgroups of transplant patients at higher risk of posttransplant complications has become a big challenge for public health, since it might improve long-term outcomes. However, until now, biomarkers of posttransplant complications have poor predictive value, rising the need to explore novel approaches for the management of transplant patient [20]. The dynamism of the epigenome and the long-lasting effect in response to environmental stimuli make the epigenetic mechanisms a suitable field of research for either biomarker discovery or the development of novel therapeutic strategies [18]. Overall, it has been acknowledged that epigenetic mechanisms play a crucial role in the multiple biological events involved in posttransplant complications, such as alloimmune response, ischemia/reperfusion (I/R) injury, and kidney graft fibrosis [18, 50]. It is also worth mentioning that both the recipient and the donor continuously undergo dynamic epigenetics modifications, even before transplantation [51, 52]. Meht and colleagues first described the usefulness of epigenetic modifications as rejection biomarkers [53]. The authors compared the methylation status of the promoters of DAPK and CALCA genes in urinary DNA from deceased or living donor kidney transplant recipients after 48 hours from the transplantation, and 65 healthy controls. CALCA hypermethylation was more frequently reported in kidney transplant recipients compared with healthy controls and, in addition, CALCA methylation was more frequent in kidney transplant recipients from deceased than from living donors. Interestingly, there was a nonsignificant trend toward CALCA hypermethylation in patients with biopsy-proven acute tubular necrosis, when compared with acute rejection and delayed or immediate graft function [53].

### 4.1. DNA Methylation and Alloimmune Response

Epigenetic mechanisms might also affect the immune response of the recipient, which is a crucial driver of the alloresponse to the graft [54–57]. Although the use of immunosuppressive drugs improved the short-term kidney graft survival and decreased the incidence of acute graft rejection, the latter still remains accountable for one-tenth of graft loss [5]. The activation of immune cells relies on integrated pathways that in turn are tightly regulated by transcription factors and chromatin remodelers [58]. As extensively reviewed by Mas and colleagues [19], the regulation of gene transcription by epigenetic mechanisms might determine cell plasticity and the strength of posttransplant immune responses.

The major histocompatibility complex (MHC) encodes glycoproteins, which present antigens to the immune system, and its expression is fundamental to alloantigen recognition. Both in physiological (i.e., gametes and embryonic cells) and in pathological (i.e., neoplastic cells) conditions, the downregulation of MHC expression confers a degree of protection from the immune system [59]. In acute rejection there is an increase in MHC II glycoproteins within the allograft and recently it has been demonstrated that DNA methylation and histone modifications affected MHC class I and II expression [60]. DNA methylation also modulates T-cell activation through the production of interleukin-2 (IL-2). In fact, the IL-2 promoter is methylated in inactive T cells, while DNA demethylation allows upregulation of IL-2 following simultaneous T-cell receptor and costimulatory signalling [61]. Most of immune cells involved in allograft rejection are influenced by epigenetic factors. Hence, the investigation of DNA methylation profiles of immune cells before and after kidney transplantation might help the discovery of novel potential biomarkers for the clinical management of patients. For instance, epigenetic mechanisms might affect transcription factors, cytokines, and other molecules that are essential to control the transcriptional profiles and functions of memory T-cell [62]. Steinfelder and colleagues demonstrated that demethylation of the CCR6 gene, which encodes for a chemokine receptor in memory CD4+ T cells, enabled the migration toward the renal proximal tubular epithelial cells [63]. Several studies also demonstrated that epigenetic mechanisms modulated the cytolytic activity of natural killer (NK) cells, which are important in promoting rejection or tolerance [64], by regulating the expression of several NK cell receptors (i.e., KIR, NCRs, and NKG2D) and cytotoxic molecules (i.e., GRZ and PRF) [65, 66]. Others reported the complex epigenetic regulatory systems that modulated the differentiation of hematopoietic stem cells into antibody-producing B cells and antibody production [67–70]. However, most of studies focused on Foxp3, which encodes the transcription factor Scurfin. Foxp3 regulates development and function of CD25+CD4+ regulatory T (T_R_) cells [71], which in turn maintain immunological self-tolerance and reduce many immune responses [72, 73]. These cells are mainly produced in the thymus as a functionally mature T-cell subpopulation specialized for immune suppression. In line with other genes, methylation of CpG sites within the Foxp3 gene leads to gene silencing whereas complete demethylation is necessary for stable and continuous Foxp3 expression [19]. Interestingly, subpopulations of T_R_ cells differ in the methylation of the T_R_-specific demethylated region within the Foxp3 gene [74]: while T_R_-specific demethylated region is methylated in naive CD4+ CD25− T cells, activated CD4+ T cells, and TGF-*β*–induced adaptive T_R_ cells, they are demethylated in natural T_R_ cells [74]. The main role of Foxp3+ natural T_R_ cells is to suppress several effectors of inflammation, such as T helper (T_H_) 1, T_H_2, and T_H_17 cells [72, 73]. As comprehensively reviewed by Wilson and colleagues, there are also several lines of evidence demonstrating that chromatin conformation and DNA methylation at lineage restricted cytokine, transcription factor genes, and their regulatory elements in T_H_ cells both reflect and affect their development and functions [75].

### 4.2. DNA Methylation and Ischemia-Reperfusion Injury

Several studies proposed that ischemia-reperfusion injury might cause DNA methylation changes in the donor organ. During the ischemic period—in several clinical settings including kidney transplantation—tissues are deprived of oxygen and nutrients required to maintain physiological metabolism and energy homeostasis [76]. In kidney transplantation, the ischemia-reperfusion injury causes a series of pathological responses ranging from inflammation and fibrosis to cell and organ graft injury [76–78]. Pratt and colleagues were the first to propose that modifications of methylated CpG sites may occur as a result of prolonged ischemia-reperfusion injury in kidney transplantation [79], which is in turn associated with chronic nephropathy posttransplantation. In a rodent model of kidney transplantation, they demonstrated that prolonged cold ischemia in rat kidneys caused demethylation of a specific CpG site within the IFN-*γ* response element resident in the promoter region of complement component 3 (C3) gene [79]. Loss of transcriptional repression of this gene contributes to provide a plausible explanation for the accentuated immunologic injury, which often follows protracted ischemia of the allograft.

### 4.3. DNA Methylation and Kidney Graft Fibrosis

Progressive interstitial fibrosis is the crucial final pathway in renal destruction in either native or transplanted kidneys. Its pathogenesis is complex and comprises both immune- and non-immune-mediated mechanisms, culminating in interstitial fibrosis, tubular atrophy, and progressive loss of graft function. Similar to wound repair, fibrosis is triggered by an injury and characterized by the deposition of extracellular matrix through activated fibroblasts but conversely it can progress even after the injury has disappeared [80]. Fibrogenesis is the result of complex interactions among the different involved cell types which is coordinated by an extensive network of growth factors and signalling pathways [81]. Mechanisms that contribute to the maintenance of the profibrotic environment have not been well elucidated, but there is emerging evidence for the effects of epigenetics on gene expression and kidney fibrogenesis. Several lines of evidence demonstrated the role of DNA methylation in an abnormal wound healing process that resulted in fibrogenesis in CKD [48, 82]. However, few preclinical studies reported that DNA methylation might activate the fibrogenesis process in the kidney [83], suggesting a possible role for oxidative stress and inflammatory cytokines [84]. A genomewide methylation study of tubule epithelial cells identified ≈5000 differentially methylated CpG sites between CKD and control patients [82]. Interestingly, functional annotation analysis revealed that most of these regions were within or near developmental and profibrotic genes and that their methylation level correlated with the expression of many profibrotic genes [85]. In addition, a genomewide methylation study of fibroblasts identified 12 genes that were hypermethylated in fibrotic but not in nonfibrotic kidney biopsies [86]. Among these genes, hypermethylation of RASAL1—encoding an inhibitor of the Ras oncoprotein—was investigated further, since its silencing led to fibroblast activation by increased Ras-GTP activity. Notably, in vivo studies demonstrated that kidney fibrosis is ameliorated in DNMT1+/− mice [86], suggesting that RASAL1 hypermethylation was mediated by DNMT1. To uncover the molecular mechanisms that characterized kidney allografts, Bontha and colleagues applied an integrative multiomics approach in biopsies collected 24 months after transplantation. The authors reported hypomethylation of CpG sites within genes involved in activation of CD8+ and CD4+ T cells and MHC genes, and hypermethylation of genes related to metabolic functions, integrity, and structure of kidney [80].

### 4.4. DNA Methylation and Other Long-Term Complications after Kidney Transplantation

Aberrant DNA methylation is also studied for predicting long-term complications after kidney transplantation. For instance, transplant recipients are more likely to develop cutaneous squamous cell carcinoma (cSCC) [87, 88], for which immunosuppressive treatment seems to be a significant risk factor. Interestingly, Sherston and colleagues investigated methylation of T_R_-specific demethylated region within the Foxp3 gene as a marker for cSCC in kidney transplant recipients [89]. The authors followed 58 survivors of a cohort of long-term kidney transplant patients, with and without skin cancer [89]. They found a significant increase in the proportion of demethylated CD4+FOXP3+ cells in patients who had previously developed cSCC. Although these results highlighted the methylation of T_R_-specific demethylated region as a potential biomarker for cSCC posttransplantation [89], the use of peripheral blood mononuclear cells instead of sorted T_R_ cells may represent a limitation of the study. More recently, Peters and colleagues aimed to determine differentially methylated regions in T cells and their role in the development of cSCC in transplant patients [90]. Before transplantation, they compared DNA methylation of T cells between 27 recipients who developed a* de novo* cSCC and 27 who did not manifest cSCC. The authors found different methylation status in regulatory genomic and bivalent enhancer regions that coded for a zinc-finger protein (i.e., ZNF577) and a protein involved in T-cell migration (i.e., FLOT1), respectively [90]. While the DNA methylation status remained relatively stable in the majority of regions, it significantly changed in 9 differentially methylated regions after transplantation [90], and this could have a long-lasting effect on posttransplant cSCC development.

Recently, the effects of epigenetic mechanisms in cellular and molecular pathways involved in the pathogenesis of cardiorenal syndromes have been proposed [91]. CKD is associated with accumulation of uremic toxins and enhanced oxidative stress, which in turn might affect epigenetic signatures, including DNA methylation. Interestingly, it has been demonstrated that global hypermethylation is independently associated with cardiovascular mortality in patients with CKD [49]. Hypertension is one of the most common complications in kidney transplant recipients, increasing the risk of graft loss and other cardiovascular diseases. In these patients, treatment with angiotensin II (Ang II) blockers for preventing or treating hypertension is closely associated with improved survival. An* in vivo* study demonstrated that DNA methylation modulated the recipient vascular Ang II receptor (AT1R) gene expression, which in turn increased the vascular contractility in response to Ang II [92]. Another complication in kidney transplant recipients is the new-onset diabetes after transplantation (NODAT), which increases the risk of cardiovascular disease, infections, graft loss, and mortality [93, 94]. A recent genomewide DNA methylation analysis of adipose tissue found no significant difference in global DNA methylation between NODAT patients and healthy controls. However, patients who developed NODAT exhibited aberrant DNA methylation in ≈ 900 regions that were involved in insulin resistance, type 2 diabetes, and inflammation. These findings suggested that changes in DNA methylation of adipose tissue might increase infiltration of immune cells with consequent insulin resistance and inflammation, in patients who finally developed NODAT [95].

## 5. Conclusions

Uncovering the clinical potential of DNA methylation in CKD and complications after kidney transplantation is one of the main challenges toward the management of kidney transplant recipients. While aberrant DNA methylation has been plenty described in CKD by* in vivo* and epidemiological studies, further research is needed to discover novel potential biomarkers for kidney transplant rejection and complications. Our review highlights the fact that research behind the role of DNA methylation in kidney transplantation has so far exclusively been in the area of basic research, using* in vitro* or* in vivo* candidate-gene studies. Few studies provided evidence that epigenetic modifications might affect the individual risk to develop posttransplant complications. Biomarker validation also offers the possibility of identifying novel therapeutic targets. In fact, epigenetic drugs—especially DNA methylating or demethylating agents—are becoming available in oncology, and their potential to maintain functions and integrity of the transplanted organ should be investigated. Furthermore, combined with routine clinical tests, the identification of biomarkers will contribute to an improvement of the patient management. Accordingly, further translational studies should be encouraged to transfer the above-mentioned knowledge to the clinic. In the forthcoming future the monitoring of DNA methylation in kidney transplant patients could become a plausible strategy toward the prevention and/or treatment of kidney transplant complications in the clinical setting and could be useful for identifying those patients who are at higher risk of developing a cardiovascular complication after transplantation, which is the leading cause of death in kidney transplant recipients.
